# Vitamin A Supplementation Alleviates Extrahepatic Cholestasis Liver Injury through Nrf2 Activation

**DOI:** 10.1155/2014/273692

**Published:** 2014-07-09

**Authors:** Guiyang Wang, Peng Xiu, Fu Li, Cheng Xin, Kewei Li

**Affiliations:** Department of Biliary-Pancreatic Surgery, Ren Ji Hospital, School of Medicine, Shanghai Jiao Tong University, Shanghai 200127, China

## Abstract

*Aim*. To investigate the role of vitamin A in liver damage induced by bile duct ligation (BDL) in rats. *Methods*. Thirty male Wistar rats were randomly divided into three groups: SHAM group, BDL group, and BDL + VitA group . The concentrations of retinol and retinyl palmitate in the liver were analyzed using HPLC, and liver function was evaluated by the level of TBIL, ALT, AST, and ALP in serum. Hepatic oxidative status was estimated by measuring T-SOD, CAT, GSH, MDA, and AOPP. Nrf2 expression was assessed using immunohistochemistry and western blotting, and EMSA was performed to determine Nrf2 DNA-binding activity. The expression of the downstream factors such as Ho1 and Nqo1 was also examined using immunohistochemistry and western blotting assays. *Results*. Vitamin A treatment restored levels of retinoids in liver, improved liver function, alleviated oxidative stress, and facilitated the translocation of Nrf2 to the nucleus in the experimental obstructive jaundice. Vitamin A was also found to increase the expression of Nrf2 downstream proteins such as Ho1 and Nqo1. *Conclusion*. Vitamin A was here found to ameliorate cholestatic liver injury. This effect may be related to the activation of Nrf2/ARE pathway in bile duct ligation rats.

## 1. Introduction

The mechanisms of hepatic damage caused by cholestasis are complicated. Recent studies have shown that reactive oxygen species (ROS) play a major role in the pathogenesis of cholestasis [1–3]. Excessive ROS generated during cholestasis can cause oxidative damage to biological macromolecules and trigger mass consumption of antioxidants and aggravated oxidative damage [2, 4]. The administration of antioxidants has been shown to exert beneficial effects in the prevention of cholestasis liver injury [5–9]. In this way, maintaining the balance between oxidation and antioxidant systems could be an important therapeutic target for cholestasis.

The nuclear factor-erythroid 2-related factor 2 (Nrf2) has been thought to exert an important role in the cellular defense against oxidative stress [10, 11]. Heme oxygenase 1 (Ho1) and NADPH-quinone oxidoreductase 1 (Nqo1) are phase II detoxification enzymes. They are regulated by the Nrf2 [12–14]. Induction of Nrf2/ARE-dependent antioxidant enzymes and phase II detoxification is one of the major cellular defense mechanisms against oxidative stresses [11, 15, 16].

Vitamin A is a micronutrient essential to a variety of physiological processes. In healthy vertebrates, the majority of vitamin A is stored in the liver, mainly in the form of retinyl palmitate [17]. Disruption of vitamin A homeostasis was associated with many diseases, particularly liver diseases [18]. It has been reported that the concentrations of vitamin A are lower in cholestatic patients even during early stages of the disease, and vitamin A supplementation was found to provide protection against hepatic damage in a BDL rat model induced by bile duct ligation [19–22]. Vitamin A has also been reported to play its protective role by inducing Nrf2 upregulation in neuronal cells [23]. However, whether vitamin A has similar effects on liver tissue is largely unknown. The aim of this study was to explore the impact of vitamin A supplementation on the prevention of cholestatic liver injury in rats and determine whether vitamin A administration activates Nrf2/ARE pathway to exert protective effects on liver damage induced by bile duct ligation.

## 2. Materials and Methods

### 2.1. Animals

Male Wistar rats (5 weeks old, weighing 190–220 g) were obtained from the Shanghai Slac Experimental Animal Centre (Shanghai, China). The rats were housed in cages, under controlled temperature (24 ± 2°C) and humidity (50 ± 10%) conditions with 12 h light/dark cycles and were given a standard diet and water ad libitum. The experiments were carried out according to the guidelines set forth by the Ethics Committee of Renji Hospital (SYXY(hu)2011-0121).

### 2.2. Experimental Design and Sample Collection

The rats were randomly assigned to three groups of 10 animals each: SHAM-operated group (SHAM), bile duct ligated group (BDL), and BDL treated with vitamin A group (BDL + VitA). Rats in the SHAM-operated group were subjected to a 1.5 cm upper midline abdominal incision, and the common bile duct was isolated from the surrounding tissues. The rats in the BDL group underwent the same procedure and the common bile duct was doubly ligated with a 4–0 silk suture and transected between the two ligatures. Vitamin A-treated rats received daily administration of retinyl acetate (Donghai Pharmaceutical Co., Ltd., Shanghai, China), namely, esters of retinol, 100,000 I.U./kg dissolved in bean oil by gavage, but those in SHAM group and BDL group were only given an equal volume of bean oil. The dose of vitamin A given to rats was selected based on information from previous reports [21]. After two weeks, rats were killed to collect blood and livers. Plasma was immediately separated by centrifugation (3000 r/min for 10 min at room temperature) and stored at −20°C. The same portion of each liver was removed and fixed in 10% neutral formalin and the remaining portion was stored at −80°C for further analysis.

### 2.3. Liver Retinol and Retinyl Palmitate Extraction and Quantification by HPLC

The sample extraction process referenced to previous methodology [24–26]. Retinol and retinyl palmitate in liver homogenates were extracted with ethanol and hexane. Then, 150 mg liver samples were homogenized in 2 mL dehydrated ethanol containing 0.1% butylated hydroxytoluene (BHT). After complete homogenization, an equal volume (2 mL) of n-hexane was added to the homogenate/ethanol-mixture and vortexed for 1 min. After centrifugation, the hexane layer was transferred to a centrifugal tube and evaporated under N_2_ at about 40°C in a water bath. The extraction manipulation was repeated twice and the merged hexane-phases were evaporated until dryness. The residue was redissolved in 100% methanol and was filtered with strainer (0.22 *μ*m). An aliquot of 20 uL was injected into the HPLC systems, which consisted of a binary HPLC pump (Waters1525) and ultra-violet absorbance detector (Waters2487) for analysis. Retinoids were separated on a reverse phase column (C18 Hypersil ODS2 4.6 mm × 200 mm, 5 um) running at 1 mL/min with the mobile phase (100% methanol) and monitored at 325 nm. Quantification was performed by relating the area of the peak to areas obtained by the analysis of known quantities of the retinol and retinyl palmitate standards and expressed as *μ*g/g wet weight. Qualitative analysis was performed with retention times of the chromatographic peaks. The retinoids standard curves were established for quantification of the retinol and retinyl palmitate. All extraction manipulations and quantification of tissues were performed under dim light.

### 2.4. Measurement of TBIL, ALT, AST, and ALP in Serum

The serum levels of total bilirubin (TBIL), alanine transaminase (ALT), aspartate transaminase (AST), and alkaline phosphatase (ALP) were measured with an automatic analyzer (HITACHI7600-20).

### 2.5. Measurement of T-SOD, GSH, CAT and MDA in Liver Homogenates

The content of total superoxide dismutase (T-SOD), glutathione (GSH), catalase (CAT), and malondialdehyde (MDA) in liver homogenate was detected with reagents kits purchased from Jiancheng Biologic Company (Nanjing, China). Manipulations were performed according to the manufacturer's protocols.

### 2.6. Measurement of Advanced Oxidation Protein Products

The concentrations of advanced oxidation protein products (AOPP) were measured by spectrophotometry as described by Witko-Sarsat et al. [27]. Liver tissue samples were homogenized by 50 mM ice-cold Tris-HCl (pH 7.4) and centrifuged at 5000 g for 10 min. Tissue supernatants were then diluted in phosphate buffered saline at a ratio of 1 : 5. The sample was mixed with potassium iodide and acetic acid, and the absorbance of the reaction mixture was immediately read at 340 nm. Chloramine was chosen as a standard and the AOPP concentrations were expressed as *μ*mol per mg protein.

### 2.7. Histological Examination and Immunohistochemical Staining

Liver tissues were fixed in 10% neutral buffered formalin, embeddedin paraffin, and sectioned. The paraffin slices were stained with hematoxylin and eosin (HE) after the treatment of deparaffinization and dehydration. For immunohistochemical analysis, hepatic sections were placed in 3% H_2_O_2_ for 10 min, followed by incubation with anti-Nrf2 (Santa Cruz, CA, USA) antibody, anti-Ho1 (Abcam, Cambridge, UK), and anti-Nqo1 (Sigma, St. Louis, USA), at 37°C for 2 h. Yellow and brown staining in the cytoplasm and nucleus were considered positive. Five microscopic fields were randomly chosen per slice, and the number of cells per field was counted.

### 2.8. Western Blotting

Nuclear and cytoplasmic proteins were extracted with nuclear and cytoplasmic extraction reagents kits (Pierce, Rockford, IL, USA) according to the manufacturer's protocol for Nrf2, Ho1, and Nqo1 detection. Protein concentrations were measured using BCA protein assay. Proteins were separated by 10% or 12% SDS-PAGE and then transferred onto PVDF membranes. The transferred membranes were blocked with TBS-T (10 mmol/L Tris-HC1, 150 mmol/L NaC1, 0.1% Tween-20) containing 5% skim milk for 1 h at room temperature. The membranes were washed in TBS-T (10 min × 3) and then the membranes were incubated overnight at 4°C with anti-Ho1 (Abcam, Cambridge, UK), anti-Nqo1 (Sigma, St. Louis, USA), anti-Nrf2 (Santa Cruz, CA, USA), anti-*β*-actin (Abmart, Shanghai, China), and anti-PCNA (Santa Cruz, CA, USA) in TBS-T. The membranes were washed three times again in TBS-T; the membranes were incubated with horseradish peroxidase conjugated to anti-rabbit IgG (Weiao Biotech Ltd, Shanghai, China) overnight at 4°C. The expression of targeted proteins was determined using Odyssey machine and optical density of band was analyzed by using Image J software.

### 2.9. Electrophoretic Mobility Shift Assay

EMSA was performed to determine Nrf2 DNA-binding activity using a commercial kit (Pierce, Rockford, IL, USA). Consensus oligonucleotide probes of Nrf2 (5′-TGGGGAACCTGTGCTGAGTCACTGGAG-3′) were labeled with biotin. Nuclear extracts were prepared using a kit (Pierce, Rockford, IL, USA). Protein concentrations were quantified using a BCA protein assay. Nuclear extract (20 *μ*g) was incubated with 20 ng of biotin-end-labeled target DNA for 30 min at room temperature in 1 × binding buffer. Assays were loaded onto native 6% polyacrylamide gels preelectrophoresed in 0.5 × Tris-borate-EDTA (TBE) for 60 min and electrophoresed at 100 V before transfer to a positively charged nylon membrane in 0.5 × TBE at 100 V for 30 min. The membrane was UV cross-linked and binding activity of Nrf2 to the probe was determined using a chemiluminescent EMSA kit (Beyotime, China).

### 2.10. Statistical Analysis

Statistical analysis was performed using SPSS version 17.0 software. Data are presented as mean ± standard deviation (SD). Analysis of variance, Duncan's test, and Dunnett's test were used to assess differences between multiple groups. *P* < 0.05 was considered statistically significant.

## 3. Results

### 3.1. General Observations

In the SHAM group, all rats survived throughout the experiment. In the BDL group, 1 died from duodenal rupture caused by operation on day 2 and 1 rat died of abdominal infection due to bile leakage on day 6 after operation. In BDL + vitA group, 1 died from primary peritonitis on day 8 after operation. Jaundice occurred in 3 days after surgery and dilatation of the remnant common bile duct proximal to the ligature was observed upon reoperation in bile duct ligated rats.

### 3.2. Morphological Studies

**Figure 1:**
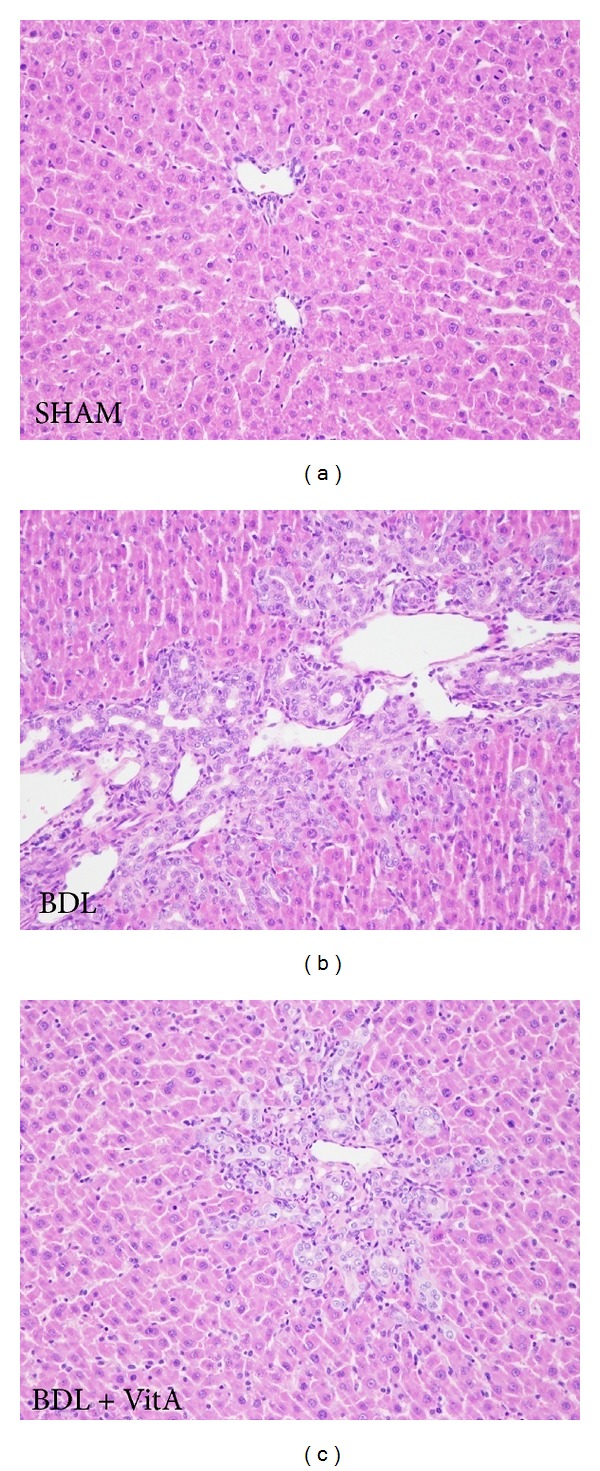
Pathomorphological changes in each group (HE, original magnification: ×200) (a): liver tissue in the SHAM group. (b): Bile duct proliferation, necrosis, and inflammatory cell infiltration were observed in the BDL group. (c): Proliferation of bile ducts, necrosis, and inflammatory cell infiltration in the BDL + vitA group was milder than in the BDL group.

Cholangiocyte hyperplasia occurred in BDL rats. Necrosis was scattered in the liver accompanied by inflammatory cells infiltrated especially around the portal areas (Figure 1(b)). However, vitamin A supplementation distinctly improved these pathological changes of the liver in BDL + vitA group relative to the BDL group (Figure 1(c)).

### 3.3. Concentrations of Retinol and Retinyl Palmitate in Liver

**Figure 2:**
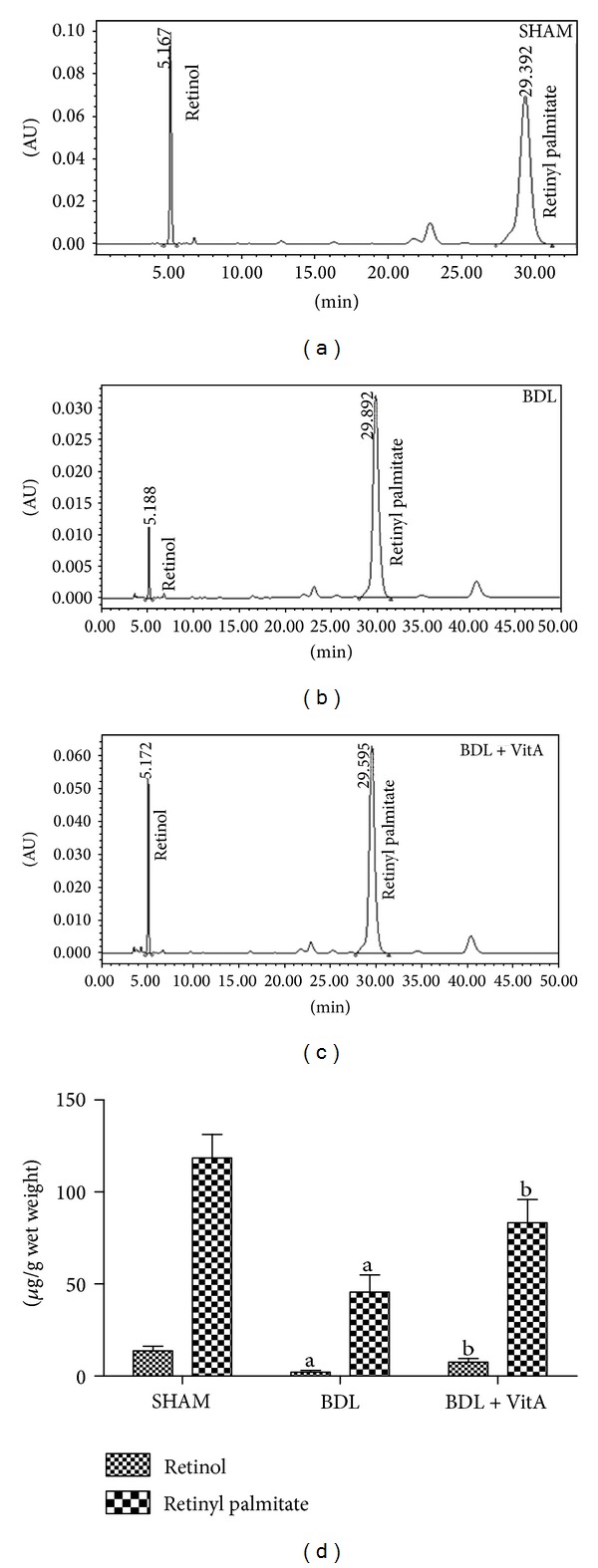
HPLC analysis of liver retinol and retinyl palmitate. (a)–(c): SHAM group (*n* = 10); BDL group (*n* = 8); BDL + vitA group (*n* = 9). Abscissa and ordinate represent the retention time (min) and absorbance (AU) of retinol and retinyl palmitate. (d): Data are presented as mean ± SD. ^a^ *P* < 0.05 versus SHAM group; ^b^ *P* < 0.05 versus BDL group.

Retinol and retinyl palmitate were eluted for 5.19 ± 0.08 min and 29.61 ± 0.29 min, respectively. Levels of retinol and retinyl palmitate in the liver tissue were lower in the BDL group (retinol: 13.65 ± 2.56 *μ*g/g wet weight versus 2.01 ± 1.05 *μ*g/g wet weight, *P* < 0.05; retinyl palmitate: 118.41 ± 12.88 *μ*g/g wet weight versus 45.53 ± 9.42 *μ*g/g wet weight, *P* < 0.05, Figure 2) and were significantly increased after vitA supplementation (retinol: 7.61 ± 2.01 *μ*g/g wet weight versus 2.01 ± 1.05 *μ*g/g wet weight, *P* < 0.05; retinyl palmitate: 83.28 ± 12.64 *μ*g/g wet weight versus 45.53 ± 9.42 *μ*g/g wet weight, *P* < 0.05, Figure 2).

### 3.4. Serum Concentrations of TBIL, ALT, AST, and ALP

**Figure 3:**
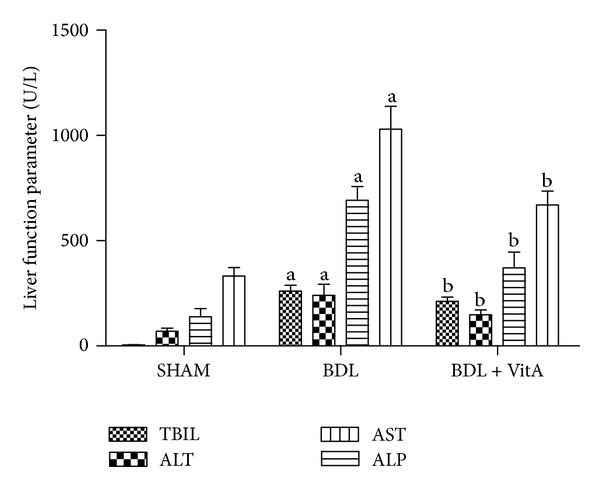
Levels of TBIL, ALT, AST, and ALP in serum. A: SHAM group (*n* = 10); B: BDL (*n* = 8); B: BDL + vitA group (*n* = 9). Data are presented as mean ± SD. ^a^ *P* < 0.05 versus SHAM group; ^b^ *P* < 0.05 versus BDL group.

The serum concentrations of* TBIL*, ALT, AST, and ALP in BDL group increased visibly compared with those in SHAM group (TBIL: 260.07 ± 28.32 *μ*mol/L versus 3.92 ± 1.43 *μ*mol/L, *P* < 0.05; ALT: 240.86 ± 52.41 U/L versus 68.77 ± 15.04 U/L, *P* < 0.05; AST: 691.68 ± 65.45 U/L versus 138.59 ± 38.40 U/L, *P* < 0.05; ALP: 1029.14 ± 108.40 U/L versus 331.50 ± 40.71 U/L, *P* < 0.05; Figure 3). The serum concentrations of TBIL, ALT, AST, and ALP were significantly lower in the BDL + vitA group (TBIL: 211.46 ± 19.83 *μ*mol/L versus 260.07 ± 28.32 *μ*mol/L, *P* < 0.05; ALT: 147.54 ± 22.97 U/L versus 240.86 ± 52.41 U/L, *P* < 0.05; AST: 370.71 ± 75.30 U/L versus 691.68 ± 65.45 U/L, *P* < 0.05; ALP: 669.60 ± 65.65 U/L versus 1029.14 ± 108.40 U/L U/L, *P* < 0.05; Figure 3).

### 3.5. Levels of T-SOD, GSH, CAT, and MDA in Liver Homogenates

T-SOD, GSH, and CAT were all less active in liver homogenates and the level of MDA was higher in the BDL group than in the SHAM group (*P* < 0.05, Table 1). However, T-SOD, GSH, and CAT were more active in liver homogenates and the level of MDA was lower in the BDL + vitA group than in the BDL group (*P* < 0.05, Table 1).

### 3.6. Changes in Levels of Advanced Oxidation Protein Products in the Liver Tissue

**Figure 4:**
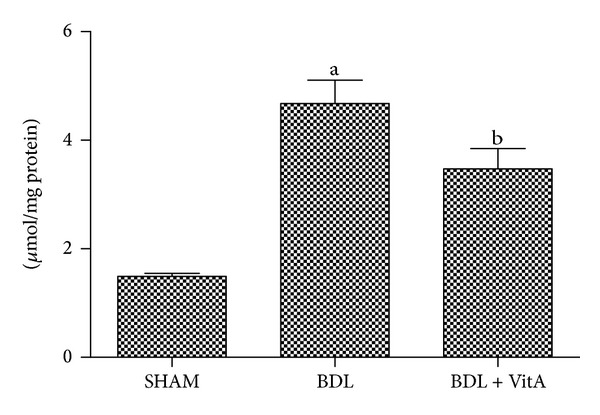
Levels of advanced oxidation protein products (AOPP) in liver tissue. A: SHAM group (*n* = 10); B: BDL (*n* = 8); C: BDL + vitA group (*n* = 9). All these values are expressed as mean ± SD.   ^a^ *P* < 0.05 versus SHAM group; ^b^ *P* < 0.05 versus BDL group.

Liver AOPP levels (*μ*mol/mg protein) were significantly higher in the BDL group than in the SHAM group (4.68 ± 0.43 versus 1.49 ± 0.58, *P* < 0.05, Figure 4), but they decreased after treatment with vitamin A (3.48 ± 0.38 versus 4.68 ± 0.43, *P* < 0.05, Figure 4).

### 3.7. Expression of Nrf2 and Downstream Proteins

**Figure 5:**
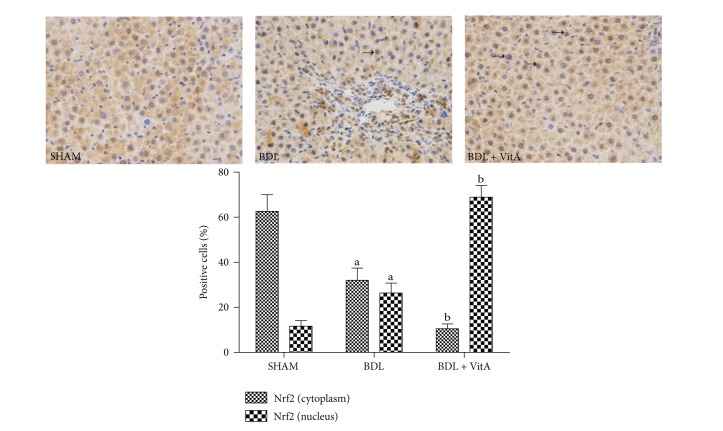
Immunohistochemical staining of liver Nrf2 expression (original magnification: ×400; arrows indicate nucleus-positive cells). More Nrf2 was concentrated in the nuclei of the BDL group than in the SHAM group, and this concentration morphology was more apparent after treatment with vitamin A.

**Figure 6:**
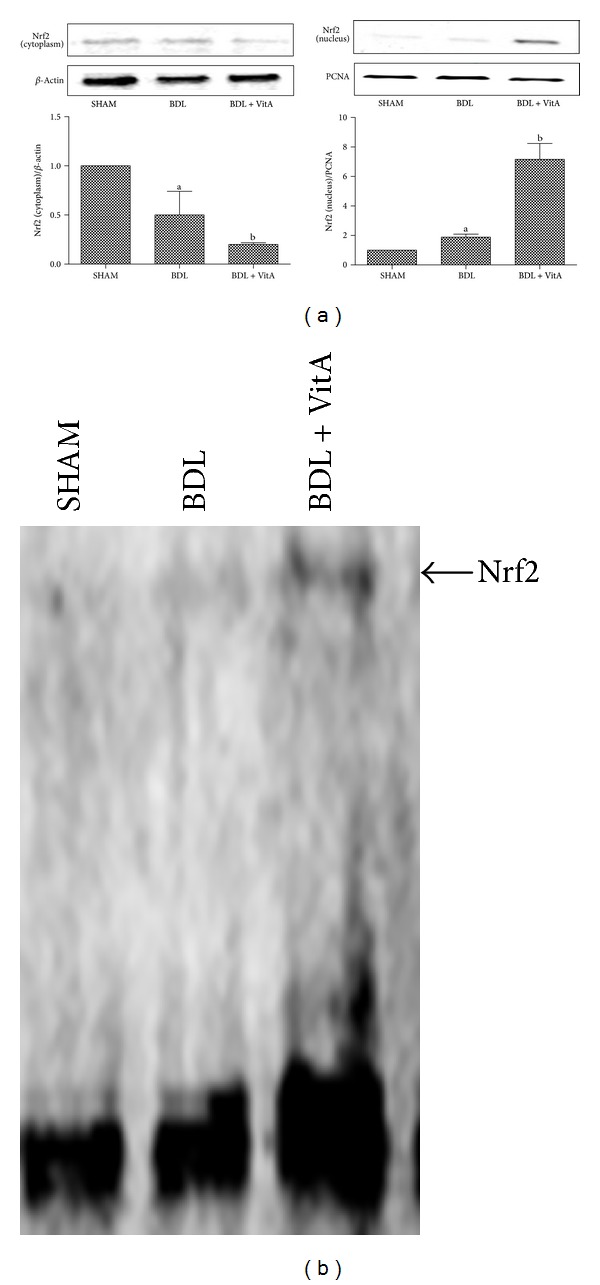
(a): Western blot results of Nrf2 expression in both the cytoplasm and nucleus from all three groups. Vitamin A significantly increased the level of Nrf2 in nucleus and decreased the level of Nrf2 in cytoplasm. PCNA and *β*-actin were used as protein loading control. (b): Gel shift assay showing Nrf2-ARE-binding activity was higher after BDL and much higher after treatment with vitamin A. SHAM group (*n* = 10), BDL group (*n* = 8), BDL + vitA group (*n* = 9). Data are presented as mean ± SD.   ^a^ *P* < 0.05 versus SHAM group; ^b^ *P* < 0.05 versus BDL group.

**Figure 7:**
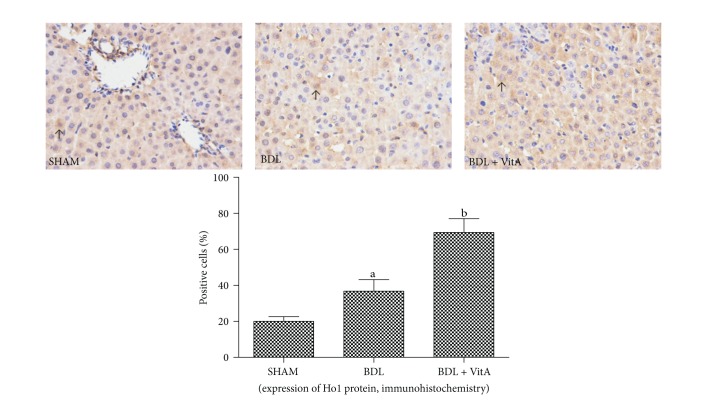
Immunohistochemical staining of liver Ho1 expression (original magnification: ×400; arrows indicate cytoplasm-positive cells). More Ho1 was expressed in the cytoplasm of the BDL group than in the SHAM group, and this morphology was more apparent after treatment with vitamin A. Data are presented as mean ± SD.   ^a^ *P* < 0.05 versus SHAM group; ^b^ *P* < 0.05 versus BDL group.

**Figure 8:**
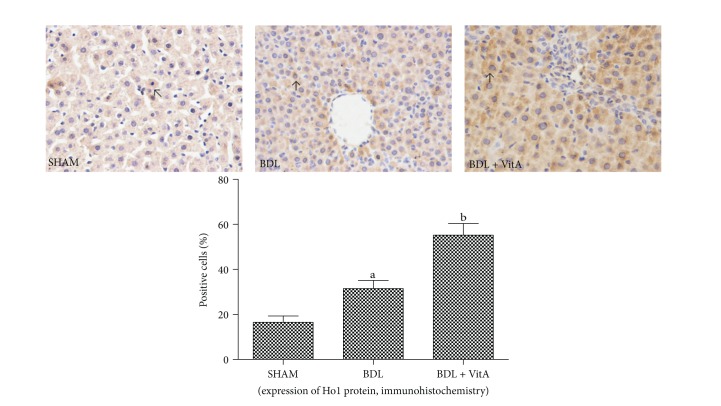
Immunohistochemical staining of liver Nqo1 expression (original magnification: ×400; arrows indicate cytoplasm-positive cells). More Nqo1 was expressed in the cytopalsm of the BDL group than in the SHAM group, and this morphology was more apparent after treatment with vitamin A. Data are presented as mean ± SD.   ^a^ *P* < 0.05 versus SHAM group; ^b^ *P* < 0.05 versus BDL group.

**Figure 9:**
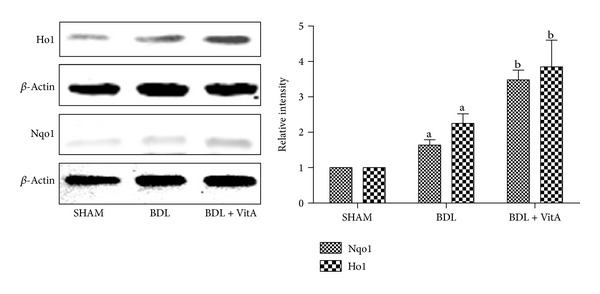
Western blot results of Ho1 and Nqo1 protein expression in the SHAM group (*n* = 10), BDL group (*n* = 8), BDL + vitA group (*n* = 9). Both Ho1 and Nqo1 proteins were upregulated after BDL, and vitamin A increased their expression in hepatic tissue further. *β*-actin was used as a loading control. Data are presented as mean ± SD.   ^a^ *P* < 0.05 versus SHAM group; ^b^ *P* < 0.05 versus BDL group.

Immunohistochemical staining and western blotting assays showed that vitamin A led to Nrf2 accumulation in nucleus in BDL rats treated with vitamin A after 2 weeks (Figure 5 and Figure 6(a)). Statistical analysis also showed there is a significant difference between Nrf2 levels in cytoplasm of the three groups (*P* < 0.05). EMSA showed Nrf2-ARE-binding activity has been enhanced after BDL and substantially increased after treatment with vitamin A (Figure 6(b)). The expression of antioxidative proteins Ho1 and Nqo1 downstream of Nrf2 was also examined. Immunohistochemistry and western blot results indicated that both Ho1 and Nqo1 proteins were upregulated after BDL (*P* < 0.05, Figures 7, 8, and 9). Additionally, administration of vitamin A for 2 weeks further increased the expression of protein overexpression in the BDL group (*P* < 0.05, Figures 7–9).

## 4. Discussion

Cholestasis is a reduction in bile flow induced by a variety of factors. It may lead to hepatocellular injury, fibrosis, cirrhosis, and, finally, liver failure. There are few effective therapies for this disease [2]. Previous studies in BDL models confirmed that vitamin A administration is effective in the repair of liver injury. Murakami et al. [21] demonstrated that enhanced expression of fibrotic markers such as keratinocyte growth factor, alpha-smooth muscle actin, and glial fibrillary acidic protein are diminished by vitamin A administration in BDL rats. He et al. [19] demonstrated that supplementation of retinoic acid with ursodeoxycholic acid can produce effects superior to those of ursodeoxycholic acid or retinoic acid treatment alone in BDL rats and that it works by reducing bile duct proliferation, necrosis, and fibrosis and liver hydroxyproline content and other inflammatory and fibrotic markers. Wang et al. [20] suggested that vitamin A derivatives such as retinoic acid inhibited liver fibrosis induced by BDL by reducing the expression of transforming growth factor-*β*1, connective tissue growth factor, and tissue inhibitors of metalloproteinase-1, diminishing the inhibition of tissue inhibitors of metalloproteinase-1 on matrix metalloproteinase-2 and matrix metalloproteinase-13 then promoting the activity of matrix metalloproteinase-2 and matrix metalloproteinase-13. However, most of the reports focused on the antifibrotic effect of vitamin A, and few reports have reported the effects of vitamin A on oxidative damage caused by cholestasis in livers.

In the present study, the concentration of vitamin A and the levels of SOD, GSH, CAT, MDA, and AOPP in hepatic tissues were used to explore the effects of vitamin A on liver oxidative damage induced by BDL. These data showed that vitamin A treatment resulted in a significant decrease in MDA and AOPP and a significant increase in SOD, GSH, and CAT levels in the livers of BDL rats by replenishing vitamin A and eventually improving liver function. This confirmed that vitamin A supplementation alleviated oxidative stress injury in livers of BDL rats.

The transcription factor Nrf2 is a key regulator of the induction of detoxifying enzymes and antioxidative stress genes [28]. Under normal conditions, Nrf2 is repressed in the cytoplasm by the actin-binding protein Kelch-like ECH associating protein-1 (Keap1). Upon oxidative stress, Nrf2 dissociates from Keap1 and translocates to the nucleus to bind to antioxidant response element (ARE), which is an enhancer element that initiates the transcription of a battery of genes encoding phase-II enzymes and antioxidant enzymes [12]. It has beenreported that Nrf2 activation counteracts cholestatic liver injury via stimulation of hepatic defense systems. After BDL, Keap1 gene-knockdown mice, which represent sustained activation of Nrf2 in the liver, showed pronounced increases in the concentrations of detoxifying enzymes and antioxidative stress genes in the livers but the number of hepatic parenchymal necrosis and the ROS remained significantly lower than in WT mice [29]. For this reason, activation of Nrf2 may be a suitable therapeutic target for prevention and amelioration of cholestatic liver injury. Data collected here showed that vitamin A exerted protection against oxidative stress induced by BDL, at least partially attributable to translocation of Nrf2 from the cytoplasm to the nucleus.

Both Ho1 and Nqo1 are easily-induced phase-II enzymes regulated by the Keap1/Nrf2/ARE pathway, and the antioxidant activity of Ho1 and Nqo1 has been confirmed [30, 31]. The present study showed that the expression and concentrations Ho1 and Nqo1 were significantly higher in the BDL group than in the control group and were even higher after vitamin A treatment (*P* < 0.05). These results indicated that vitamin A promoted the activation of Nrf2 and its target Ho1 and Nqo1 in liver tissue of BDL rats. This may be associated with the improvement of hepatic injury induced by oxidative stress in BDL rats. The protective effects of vitamin A may involve multiple mechanisms and more detailed study is needed to identify the true mechanism.

## 5. Conclusions

In conclusion, these results suggest that the concentration of vitamin A is lower in BDL rats than in control rats but is restored after supplementation. Vitamin A activated Nrf2/ARE signaling and promoted antioxidant mechanisms in BDL rats. This was characterized by improvements in liver tissue pathology and liver function, lower levels of peroxidation products, and an enhanced antioxidant capacity. This was consistent with the expression and changes in the concentrations of Nrf2, Ho1, and Nqo1. Vitamin A supplementation may not only improve vitamin A status, but also exerts a role of Nrf2 activators and alleviates cholestasis liver injury in BDL rats. This may open new perspectives for therapeutic uses of vitamin A in patients with cholestasis.

## Figures and Tables

**Table 1:** Levels of SOD, GSH, CAT, and MDA in hepatic tissues (mean ± SD).

Group	*n*	T-SOD (U/mgprot)	GSH (mg/gprot)	CAT (U/mgprot)	MDA (nmol/mgprot)
SHAM	10	222.70 ± 9.39	4.92 ± 0.19	113.31 ± 9.68	0.81 ± 0.11
BDL	8	176.49 ± 10.79^a^	3.50 ± 0.14^a^	71.46 ± 4.84^a^	3.08 ± 0.87^a^
BDL + vitA	9	202.68 ± 5.82^b^	4.23 ± 0.32^b^	82.39 ± 4.00^b^	2.07 ± 0.14^b^

^a^ *P* < 0.05 versus SHAM group; ^b^ *P* < 0.05 versus BDL group.

## References

[1] Roma MG, Sanchez Pozzi EJ (2008). Oxidative stress: a radical way to stop making bile. *Annals of Hepatology*.

[2] Copple BL, Jaeschke H, Klaassen CD (2010). Oxidative stress and the pathogenesis of cholestasis. *Seminars in Liver Disease*.

[3] Anthérieu S, Azzi PB, Dumont J (2013). Oxidative stress plays a major role in chlorpromazine-induced cholestasis in human HepaRG cells. *Hepatology*.

[4] Jung K, Kwak M (2010). The Nrf2 system as a potential target for the development of indirect antioxidants. *Molecules*.

[5] Barón V, Muriel P (1999). Role of glutathione, lipid peroxidation and antioxidants on acute bile-duct obstruction in the rat. *Biochimica et Biophysica Acta*.

[6] Soylu AR, Umit H, Tezel A (2006). Antioxidants vitamin E and C attenuate hepatic fibrosis in biliary-obstructed rats. *World Journal of Gastroenterology*.

[7] Tain YL, Chen CC, Lee CT (2013). Melatonin regulates L-Arginine transport and NADPH oxidase in young rats with bile duct ligation: role of protein kinase C. *Pediatric Research*.

[8] Tokac M, Taner G, Aydin S (2013). Protective effects of curcumin against oxidative stress parameters and DNA damage in the livers and kidneys of rats with biliary obstruction. *Food and Chemical Toxicology*.

[9] Galicia-Moreno M, Favari L, Muriel P (2012). Antifibrotic and antioxidant effects of N-acetylcysteine in an experimental cholestatic model. *European Journal of Gastroenterology and Hepatology*.

[10] Nguyen T, Nioi P, Pickett CB (2009). The Nrf2-antioxidant response element signaling pathway and its activation by oxidative stress. *Journal of Biological Chemistry*.

[11] Niture SK, Kaspar JW, Shen J, Jaiswal AK (2010). Nrf2 signaling and cell survival. *Toxicology and Applied Pharmacology*.

[12] Zhang DD (2006). Mechanistic studies of the Nrf2-Keap1 signaling pathway. *Drug Metabolism Reviews*.

[13] Chen C, Pung D, Leong V (2004). Induction of detoxifying enzymes by garlic organosulfur compounds through transcription factor Nrf2: effect of chemical structure and stress signals. *Free Radical Biology and Medicine*.

[14] Jeong W, Jun M, Kong AT (2006). Nrf2: a potential molecular target for cancer chemoprevention by natural compounds. *Antioxidants and Redox Signaling*.

[15] Das BN, Kim Y, Keum Y (2013). Mechanisms of Nrf2/keap1-dependent phase II cytoprotective and detoxifying gene expression and potential cellular targets of chemopreventive isothiocyanates. *Oxidative Medicine and Cellular Longevity*.

[16] Kaspar JW, Niture SK, Jaiswal AK (2009). Nrf2:INrf2 (Keap1) signaling in oxidative stress. *Free Radical Biology and Medicine*.

[17] Blaner WS, O'Byrne SM, Wongsiriroj N (2009). Hepatic stellate cell lipid droplets: a specialized lipid droplet for retinoid storage. *Biochimica et Biophysica Acta*.

[18] Venu M, Martin E, Saeian K, Gawrieh S (2013). High prevalence of vitamin A deficiency and vitamin D deficiency in patients evaluated for liver transplantation. *Liver Transplantation*.

[19] He H, Mennone A, Boyer JL, Cai S (2011). Combination of retinoic acid and ursodeoxycholic acid attenuates liver injury in bile duct-ligated rats and human hepatic cells. *Hepatology*.

[20] Wang H, Dan Z, Jiang H (2008). Effect of all-trans retinoic acid on liver fibrosis induced by common bile duct ligation in rats. *Journal of Huazhong University of Science and Technology-Medical Science*.

[21] Murakami K, Kaji T, Shimono R (2011). Therapeutic effects of vitamin A on experimental cholestatic rats with hepatic fibrosis. *Pediatric Surgery International*.

[22] Floreani A, Baragiotta A, Martines D, Naccarato R, D'Odorico A (2000). Plasma antioxidant levels in chronic cholestatic liver diseases. *Alimentary Pharmacology and Therapeutics*.

[23] Zhao F, Wu T, Lau A (2009). Nrf2 promotes neuronal cell differentiation. *Free Radical Biology and Medicine*.

[24] Carvalho O, Gonçalves C (2013). Retinol and retinyl palmitate in foetal lung mice: sexual dimorphism. *Critical Care Research and Practice*.

[25] Rasmussen M, Blomhoff R, Helgerud P, Solberg LA, Berg T, Norum KR (1985). Retinol and retinyl esters in parenchymal and nonparenchymal rat liver cell fractions after long-term administration of ethanol. *Journal of Lipid Research*.

[26] Schäffer MW, Roy SS, Mukherjee S (2010). Qualitative and quantitative analysis of retinol, retinyl esters, tocopherols and selected carotenoids out of various internal organs from different species by HPLC. *Analytical Methods*.

[27] Witko-Sarsat V, Friedlander M, Capeillère-Blandin C (1996). Advanced oxidation protein products as a novel marker of oxidative stress in uremia. *Kidney International*.

[28] Keum YS (2012). Regulation of Nrf2-mediated phase II detoxification and anti-oxidant genes. *Biomolecules & Therapeutics*.

[29] Okada K, Shoda J, Taguchi K (2009). Nrf2 counteracts cholestatic liver injury via stimulation of hepatic defense systems. *Biochemical and Biophysical Research Communications*.

[30] Dinkova-Kostova AT, Talalay P (2010). NAD(P)H:quinone acceptor oxidoreductase 1 (NQO1), a multifunctional antioxidant enzyme and exceptionally versatile cytoprotector. *Archives of Biochemistry and Biophysics*.

[31] Martin D, Rojo AI, Salinas M (2004). Regulation of heme oxygenase-1 expression through the phosphatidylinositol 3-kinase/ Akt pathway and the Nrf2 transcription factor in response to the antioxidant phytochemical carnosol. *The Journal of Biological Chemistry*.

